# Bergamot Essential Oil Attenuates Anxiety-Like Behaviour in Rats

**DOI:** 10.3390/molecules22040614

**Published:** 2017-04-11

**Authors:** Laura Rombolà, Laura Tridico, Damiana Scuteri, Tsukasa Sakurada, Shinobu Sakurada, Hirokazu Mizoguchi, Pinarosa Avato, Maria Tiziana Corasaniti, Giacinto Bagetta, Luigi Antonio Morrone

**Affiliations:** 1 Department of Pharmacy, Health and Nutritional Sciences, Section of Preclinical and Translational Pharmacology, University of Calabria, 87036 Rende, Italy; laura.rombola@unical.it (L.R.); lauratridico@libero.it (L.T.); damianascuteri@gmail.com (D.S.); g.bagetta@unical.it (G.B.); 2 First Department of Pharmacology, Daiichi College of Pharmaceutical Sciences, 815-8511 Fukuoka, Japan; tsukasa@daiichi-cps.ac.jp; 3 Department of Physiology and Anatomy, Tohoku Pharmaceutical University, 981-8558 Sendai, Japan; sakura@tohoku-pharm.ac.jp (S.S.); mizo@tohoku-mpu.ac.jp (H.M.); 4 Department of Pharmacy-Drug Sciences, University of Bari Aldo Moro, IT-70125 Bari, Italy; pinarosa.avato@uniba.it; 5 Department of Health Sciences, University “Magna Graecia” of Catanzaro, 88100 Catanzaro, Italy; mtcorasa@unicz.it

**Keywords:** bergamot essential oil, diazepam, behavioural tests, rat

## Abstract

Preclinical studies have recently highlighted that bergamot essential oil (BEO) is endowed with remarkable neurobiolological effects. BEO can affect synaptic transmission, modulate electroencephalographic activity and it showed neuroprotective and analgesic properties. The phytocomplex, along with other essential oils, is also widely used in aromatherapy to minimize symptoms of stress-induced anxiety and mild mood disorders. However, only limited preclinical evidences are actually available. This study examined the anxiolytic/sedative-like effects of BEO using an open field task (OFT), an elevated plus-maze task (EPM), and a forced swimming task (FST) in rats. This study further compared behavioural effects of BEO to those of the benzodiazepine diazepam. Analysis of data suggests that BEO induces anxiolytic-like/relaxant effects in animal behavioural tasks not superimposable to those of the DZP. The present observations provide further insight to the pharmacological profile of BEO and support its rational use in aromatherapy.

## 1. Introduction

**Figure 1:**
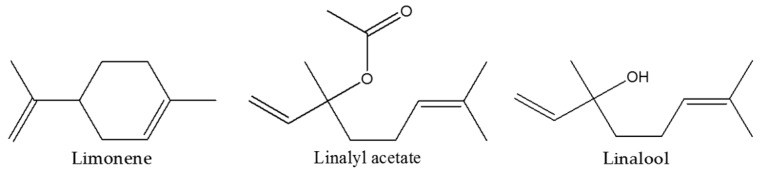
Main compounds present in volatile fraction of bergamot essential oil (BEO).

Bergamot essential oil (BEO) is obtained by cold pressing of the epicarp and part of the mesocarp of *Citrus bergamia* Risso and Poiteau, belonging to the Rutaceae family, genus Citrus. Bergamot is defined as a hybrid of bitter orange (*Citrus aurantium* L.) and lemon (*Citrus limon* L.) by some authors, or of *Citrus* *aurantium* L. and *Citrus aurantifolia Swing* by others [1]. BEO consists of several substances among which terpens and oxygenated molecules in the volatile fraction (93%–96% of total), coumarines and psoralens in the non-volatile fraction (4%–7% of total) of the essence [2,3]. Particularly, the volatile fraction includes monoterpene and sesquiterpene hydrocarbons (e.g., d-limonene, β-bisabolene, γ-terpinene, α- and β-pinene, sabinene, β-myrcene, terpinolene, and geranyl acetate) and oxygenated derivatives (e.g., linalool, linalyl acetate, neral, geranial, neryl acetate, and geranyl acetate). The most abundant compounds are the monoterpene hydrocarbons d-limonene (25.62%–53.19% of the whole essential oil), the monoterpene ester, linalyl acetate (15.61%–40.37%), and the monoterpene alcohol, linalool (1.75%–20.26%) (Figure 1) [4]. Bergamot essential oil is widely used in the cosmetic industry as perfume fixative and as an aroma in the food trade and pharmaceutical industry. As reported by the European Medicines Agency (EMA) monograph in the Herbal Medicinal Products section, traditional and folk medicine use of BEO has long been known of, but only in the last ten years have preclinical studies provided several data to support potential therapeutic use of the essential oil. BEO is used to facilitate wound healing as antiseptic and antihelminthic, effects that are supported by antimicrobial [5] and antifungal [6] activities as well as the capacity of the phytocomplex to increase oxidative metabolism in human polymorphonuclear leukocytes [7]. Furthermore, data accumulated in the literature so far indicate that bergamot oil is endowed with notable neurobiolological effects (see [8,9]) originating, at least in part, by an interference with basic mechanisms finely tuning synaptic plasticity under physiological [10,11] as well as pathological conditions, i.e., brain ischemia [12] and pain [13,14,15,16,17,18], induced under controlled and broadly validated experimental settings. Interestingly, BEO also showed neuroprotective effects in human neuroblastoma cell line [19,20,21,22]. Moreover, this phytocomplex, as with other essential oils [23,24], is widely used in aromatherapy (a branch of herbal medicine) to relieve symptoms of stress-induced anxiety [25], although limited preclinical data are actually available [26,27]. In this regard, to further characterize the neurobiological profile of the phytocomplex and, consequently, to contribute to a rational use of BEO in aromatherapy, this study investigated the effects of the essential oil in behavioural tests usually performed to study anxiolytic, stimulant or sedative and antidepressive effects of compounds as the open field (OFT) [28], elevated plus-maze (EPM) [29], and forced swimming (FST) [30,31] tasks. The results obtained with BEO are compared with the benzodiazepine diazepam (DZP).

## 2. Results

### 2.1. Effects of BEO on Open Field Test (OFT)

**Figure 2:**
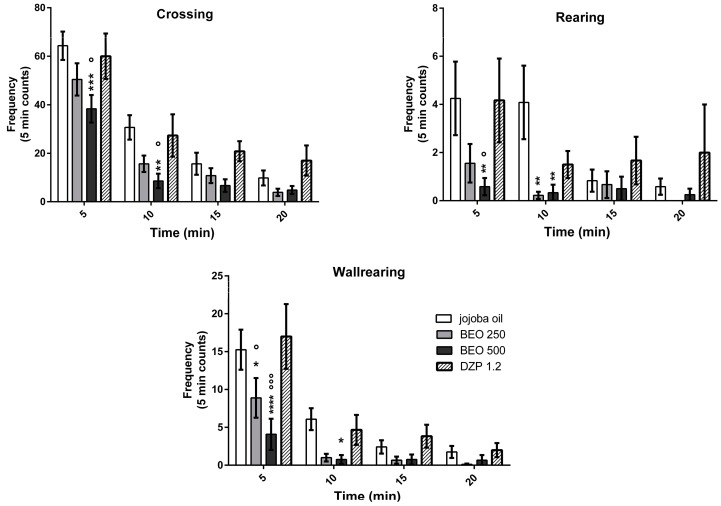
Crossing, Rearing and Wallrearing frequency in open field test in male Wistar rats after systemic (i.p.) administration of BEO (250, 500 µL/kg), benzodiazepine diazepam (DZP) (1.2 mg/kg), or jojoba oil (500 µL/kg). Data are expressed as mean ± SEM of total frequency counts in 5 min and total time in s (*n* = 6–12 per group). * *p* < 0.05, ** *p* < 0.01, *** *p* < 0.001, **** *p* < 0.0001 vs. jojoba oil group; ° *p* < 0.05, °°° *p* < 0.001 vs. DZP treated rats (two-way analysis of variance (ANOVA), followed by Tukey Multiple Comparison’s test).

Analysis of data indicates that systemic administration of bergamot essential oil (250 or 500 μL/kg) induces significant differences in the frequencies of crossing (F treatment, Ftr(3, 140) = 11.98, *p* < 0.0001, F time, Ft(3, 140) = 68.78, *p* < 0.0001, Ftxtr(9, 140) = 1.004, *p* < 0.4395), rearing (Ftr(3, 140) = 6.348, *p* = 0.0005, Ft(3, 140) = 5.249, *p* < 0.0018, Ftxtr(9, 140) = 1.610, *p* = 0.1179) and wallrearing (Ftr(3, 140) = 11.24, *p* < 0.0001, Ft(3, 140) = 32.80, *p* < 0.0001, Ftxtr(9, 140) = 2.42, *p* = 0.0138) (Figure 2) versus jojoba oil (vehicle) or DZP (1.2 mg/kg) group. Particularly, the rats treated with the higher dose of BEO (500 µL/kg) show a decrease of crossing, rearing, and wallrearing that reach statistical significance at the first two intervals of testing (5 and 10 min) versus jojoba oil treated rats (Figure 2). Interestingly, a statistically significant difference is also observed at 5 min between BEO (500 µL/kg) and DZP groups (Figure 2). Conversely, DZP does not induce statistically significant changes in these parameters compared to vehicle group.

**Figure 3:**
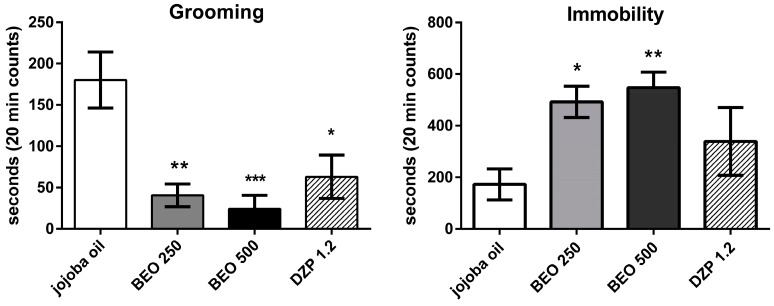
Grooming and Immobility time in the open field test in male Wistar rats after systemic (i.p.) administration of BEO (250, 500 µL/kg), DZP (1.2 mg/kg), or jojoba oil (500 µL/kg). Data are expressed as mean ± SEM of seconds (20 min counts) (*n* = 6–12 per group). * *p* < 0.05, ** *p* < 0.001, *** *p* < 0.0001 vs. jojoba oil group (one-way ANOVA, followed by Tukey Multiple Comparison’s test).

Significant differences are also shown across the groups with regard to grooming (F(3, 35) = 9.073 *p* < 0.0001) and immobility (F(3, 35) = 6.511 *p* < 0.013) behaviour (Figure 3). Particularly, a statistically significant decrease is observed for grooming in the animals treated both with BEO (250 or 500 µL/kg) or DZP (1.2 mg/kg) compared to vehicle group, while immobility is increased in the rats treated with the phytocomplex only (Figure 3).

Interestingly, at variance with the effects of BEO, the animals treated with the sedative dose of DZP (5 mg/kg), placed by the operator at the center of the arena, barely move to the periphery and remain in the same position for the entire duration of the test (data not shown).

### 2.2. Effects of BEO on Elevated Plus-Maze Test (EPM)

**Figure 4:**
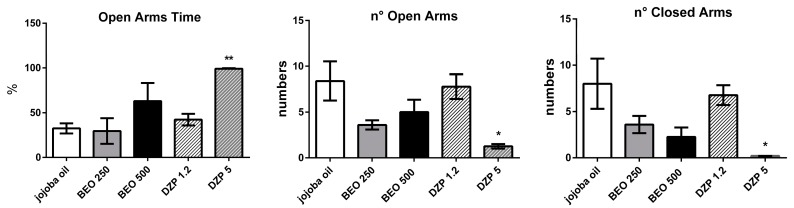
Percentage of time spent in open arm, number of open and closed arm entries in the elevated plus-maze test in male Wistar rats after systemic (i.p.) administration of BEO (250, 500 µL/kg), DZP (1.2, 5 mg/kg), or jojoba oil (500 µL/kg). Data are expressed as mean ± SEM (*n* = 4–9 per group). * *p* < 0.05, ** *p* < 0.01 vs. jojoba oil group (one-way ANOVA, followed by Tukey Multiple Comparison’s test).

In freely moving rats the administration of BEO (500 μL/kg) or DZP (1.2 mg/kg) shows a trend toward an increase of the time spent in open arms when compared to vehicle group. One way ANOVA analysis reveals statistically significant changes by DZP (5 mg/kg) only (F(4, 22) = 6.186, *p* = 0.0017). Statistically differences are also observed in the number of entries in open arms (F(4, 22) = 3.916, *p* = 0.015) or in closed arms (F(4, 22) = 4.328, *p* = 0.0098) (Figure 4).

Interestingly, at variance with the effects of BEO, the animals treated with the sedative dose of DZP, placed by the operator at the center of maze, remain in the same position for most of the duration of the task.

### 2.3. Effects of BEO on Forced Swimming Test (FST)

**Figure 5:**
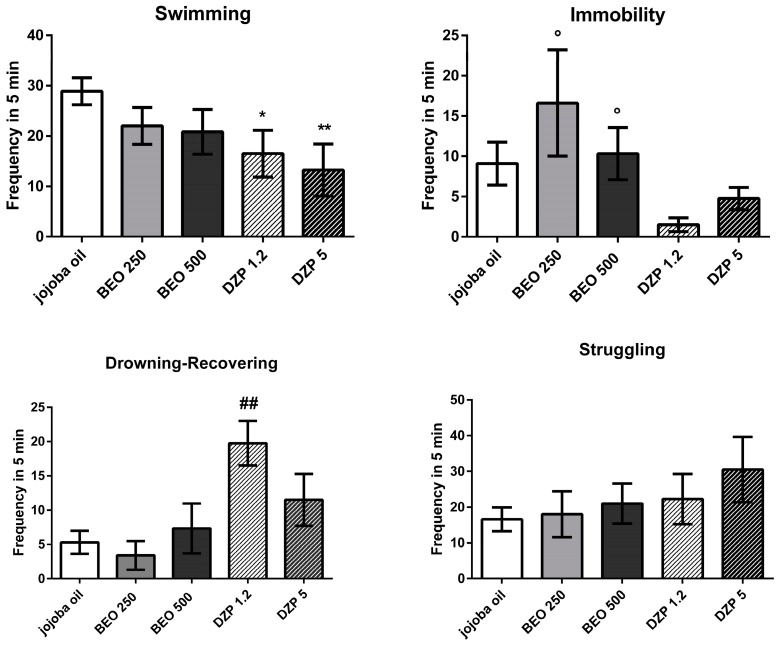
Swimming, Immobility, Drowning-Recovering and Struggling frequency in male Wistar rats after systemic (i.p.) administration of BEO (250, 500 µL/kg), DZP (1.2, 5 mg/kg), or jojoba oil (500 µL/kg). Data are expressed as mean ± SEM (*n* = 4–10 per group). * *p* < 0.05, ** *p* < 0.001 vs. jojoba oil group (unpaired *t*-test); ° *p* < 0.05 vs. DZP 1.2 mg/kg (unpaired t-test); ## *p* < 0.001 vs. jojoba oil or BEO 250 µL/kg group, respectively (one-way ANOVA, followed by Tukey Multiple Comparison’s test).

Analysis of data shows differences between BEO and DZP in FST parameters. Particularly, a trend towards a decrease in swimming is observed after both treatments that reaches statistical significance only when DZP (1.2 or 5 mg/kg) is compared to jojoba oil group (*p* = 0.016; *p* = 0.0062, respectively). Moreover, statistical analysis indicates a significant increase in immobility behaviour in rats treated with BEO (250 or 500 μL/kg) (*p* = 0.042; *p* = 0.032, respectively) versus DZP (1.2 mg/kg) group (Figure 5). In addition, drowning-recovering frequency is significantly increased (F(4, 24) = 4.53, *p* = 0.007) in DZP (1.2 mg/kg) treated rats when compared to jojoba oil or BEO (250 μL/kg) groups (Figure 5). No statistically significant difference is observed in struggling behaviour (F(4, 24) = 0.82, *p* = 0.53).

The animals treated with the dose of BEO of 100 μL/kg do not show difference versus control treated rats in any of the performed tests (data not shown).

## 3. Discussion

Essential oils are extensively used in aromatherapy in mild mood disorders and to minimize symptoms of stress-induced anxiety [23,24]. Accordingly, preclinical studies indicate that essential oils belonging to Lavander [32,33,34] and Citrus [35,36,37] species induce anxiolytic, sedative, and antidepressant effects in behavioural analyses, using the EPM, OFT, and FST tasks. Likewise, BEO is used to relieve symptoms of stress-induced anxiety (see [25]), though limited preclinical data are available [26,27].

The present study shows that the administration of BEO induces anxiolytic-like effects in animal behavioural tasks. Particularly, as with DZP, in an open field task, the phytocomplex significantly reduces grooming behaviour compared to vehicle group. During a low-stress situation grooming is a body care ritual while in stress-evoked situation it is characterized by frequent bursts of rapid short grooming activity with abnormal progression and frequent incomplete and interrupted bouts [38]. Typically, anxiolytic drugs decrease grooming in OFT [39]. BEO also decreases rearing, wallrearing, and locomotor activity compared to vehicle group. In particular, these parameters are related to attempts to escape from a novel environment and represent an important anxiety-related behaviour [40]. Conversely to BEO, rearing, wallrearing, and locomotor activity are not modified by anxiolytic dose of DZP. Interestingly, bergamot oil also increases immobility, suggesting a sedative effect in open field task. Nevertheless, in rats treated with BEO, motor activity (crossing frequency) is still detectable in the last minutes of OFT session, whereas the animals treated with the sedative dose of DZP are not vigilant and active in all OFT sessions. In fact, after DZP treatment, rats placed by the operator at the center of the arena, barely move to the periphery and remain in the same position for all the behavioural task. These data suggest that the behavioural effects of BEO in OFT are not superimposable to those of DZP.

Anxiolytic-like effects of BEO are also supported by EPM data. However, behavioural differences are observed between BEO and DZP treatments. Particularly, the higher dose of BEO increases the time spent in open arms, but the number of entries in both open and closed arms are reduced. These results seem to suggest a decrease in motor activity but not motor impairment since the same number of entries is observed in both arms. Interestingly, animals treated with the sedative dose of DZP remain in the same arm and consequently the number of entries in both arms are significantly reduced compared to vehicle group.

To gain more insight regarding locomotor differences observed after BEO and DZP treatments, this study used a forced swimming test (FST). FST is usually recognized as a model for assessing antidepressant activity of drugs, but in the literature it is also used to evaluate stimulant or sedative effects after exposure to essential oils [31].

In FST, swimming behaviour is reduced by both treatments, but a statistically significant difference is observed after DZP only. Unexpectedly, DZP induced a decrease of immobility compared to vehicle group. This apparent discrepancy could be explained by considering the drowning recovering parameter that highlights the inability of rats to stay afloat and that differs from immobility behaviour. In fact, animals treated with DZP show an increase of drowning recovering parameter, then quickly become tired and hardly able to continue to swim properly. On the contrary, BEO shows an increase in immobility behaviour compared to both jojoba oil and DZP groups. In FST, the animal is unable to touch the bottom of the cylinder. Some authors consider immobility as an adaptive response that increases the chance of the animal to survive [41,42,43]. Accordingly, the increase of immobility behaviour observed in the rats treated with BEO could be interpreted as an adaptive response to stress and represents a form of successful coping rather than failure to cope. Moreover, FST data analysis also indicates that both BEO and DZP do not show antidepressant-like effect in this task.

Altogether, these results suggest that BEO induces anxiolytic-like/relaxant effects not superimposable to those of the DZP in animal behavioural tasks. Particularly, BEO induces relaxant effects in rats, although the animals are still vigilant, and these are at variance with the effects of DZP. Spontaneous behaviour is reflected in the electroencephalographic (EEG) activity and it is known that hippocampal rhythmic slow activity and cortical low voltage fast activity dominate the EEG during “voluntary movements” but not during sedation [44]. Incidentally, previous results indicated that systemic doses of BEO increase alpha EEG frequency, correlate to relaxation, and beta brainwave activity, associated with being alert and awake [11]. A different EEG pattern is observed with DZP that decreases delta and alpha and increases beta-3/gamma activity [45,46,47]. Therefore, both behavioural and EEG data seem to support the hypothesis that other neurotransmitter systems, in addition to the GABAergic, could be likely involved in the anxiolytic/relaxant effect of bergamot oil. Komiya and colleagues (2006) observed that lemon oil vapor inhalation causes an anti-stress effect by modulating serotonergic and dopaminergic in addition to GABAergic systems in mice [35]. More recently, Chioca and colleagues (2013) and Takahashi and colleagues (2014) also showed that serotonergic neurotransmission is involved in the pharmacological mechanism by which lavender essential oil exerts its anxiolytic-like effect [34,48]. Interestingly, linalool, one of the major components of lavender oil (but also of BEO), has been the compound most linked to the anxiolytic effect of lavender [49,50,51]. Furthermore, Takahashi and colleagues suggested that linalyl acetate works synergistically with linalool and that the presence of both is essential for the whole oil to work [51]. Results by Siok and colleagues (2012) also support the role of mGlu2/3 receptor activators as potential anxiolytic compounds [47]. Incidentally, the authors of the present study previously demonstrated that BEO may interfere with mechanisms controlling synaptic levels of glutamate and other neurotransmitters in rodents [10,12]. Altogether, these data suggest that complex mechanisms may be likely implicated in BEO effects and these deserve further investigation. However, the present observations provide further insight to the neuropharmacological profile of BEO and support its rational use in aromatherapy in symptoms of stress-induced anxiety. This is of particular interest since drugs used in the treatment of chronic disabling diseases such as anxiety and mood disorders are often associated with severe side effects that adversely affect patient compliance. In particular, chronic benzodiazepines use induces drowsiness, lethargy, dizziness, vertigo, sedation, tolerance and dependence (see [52]). Over the past years, all these side effects induced patients to benefit from complementary medicines including aromatherapy [53,54,55].

Incidentally, benzodiazepines are also widely used to control disruptive behaviour and sleep disturbances in patients with dementia, though limited evidence exists for their clinical efficacy [52,56]. In this regard, aromatherapy has recently received great interest within the field of dementia treatment and the use of essential oils is increasing [57]. Ballard and colleagues (2009) reported that aromatherapy is useful in the management of agitation and aggression associated with Alzheimer disease [58].

Untreated pain is a major contributor to reduced quality of life and disability in dementia patients, and can lead to increased behavioural and psychological symptoms of dementia (BPSD) [59,60]. Therefore, the behavioural effects reported herein in conjunction with its established analgesic properties [13,14,15,16,17,18] could provide the rational basis to the use of BEO at reducing BPSD associated with dementia.

## 4. Materials and Methods

### 4.1. Animals

Male Wistar rats (250–300 g; Charles River, Calco, Italy) were used, housed at constant temperature (22 ± 1 °C) and relative humidity (50%) under a regular light-dark schedule (lights on 7 a.m. to 7 p.m.). Food and water was freely available. All experiments were carried out in accordance with the European Community Council Directive of 24 November 1986 (86/609/EEC) and in compliance with L.D. 4 March 2014 No. 26 for minimizing animal suffering and to use only the number of animals necessary to produce reliable results.

### 4.2. Bergamot Essential Oil

This study used the ‘‘whole’’ bergamot essential oil in a form that is marketed for human personal and therapeutic use. BEO was kindly provided by “Capua Company1880 S.r.l.,” Campo Calabro, Reggio Calabria (Italy) and chromatographic results on the certificate of analysis confirm that the essential oil of bergamot contained d-limonene, 39.60%; linalyl acetate, 31.09%; linalool, 9.55%. Jojoba oil (vehicle of BEO) was provided by “Company Farmalabor,” Canosa of Puglia (Italy) and DZP by pharmaceutical industry Roche S.p.A., Monza (Italy).

### 4.3. Experimental Procedure

The animals, transferred in a clean cage, were allowed to acclimatize in the behavioural room for 2 h and randomly assigned to the experimental groups. The rats were pretreated systemically (intraperitoneally, i.p.) with BEO (100, 250, or 500 μL/kg) [10,11,12], DZP (1.2 or 5 mg/kg) [37,40,61] or jojoba oil (500 μL/kg) [13,15,16] 30 min before each test. For the lowest doses of bergamot oil the total volume injected was 500 μL/kg by adding jojoba oil, an unscented oil used as vehicle. A closed circuit camera was mounted vertically above test apparatus and the rat was observed from a monitor in an adjacent room. Behavioural sessions performed between 09.00 and 17.00 A.M., during the light phase of the circadian rhythms of the animal, were videotaped for further analysis by a trained observer who remained blind to treatments. At the end of the experiment, the rat was euthanized by an overdose of isoflurane.

### 4.4. Open Field Test

The apparatus consisted of a circular arena (75 cm diameter) made of dark plastic under dim lighting (20 lux) as previously described by Walsh and Cummins (1976) [28]. Test, lasting 20 min, started by placing the rat into the center of the arena. To assess general locomotor activity, the following behavioural parameters (expressed as frequency on 5 min counts) were scored: number of square limit crossings with both forepaws, rearing (standing with the body inclined vertically, forequarters raised), and wallrearing (standing on the hind-limbs and touching the walls of the apparatus with the forelimbs). To investigate anxiety-related behaviour, time spent performing general grooming activity consisting of face grooming (strokes along the snout), head washing (semicircular movements over the top of the head and behind the ears), and body grooming (body fur licking) was measured [28]. Moreover, immobility was measured (time spend by the animal to make no movement with the body, paws, tail, and head). The scoring was performed using a video-tracking motion analysis system (Labehaviour).

### 4.5. Elevated Plus Maze Test

The experimental procedure was performed according to Pellow et al. [29]. Briefly, the Plexiglass apparatus consists of two opposite open arms (50 × 10 cm) and two closed arms (50 × 10 × 40 cm) extending horizontally at right angles from a central area (10 × 10 cm) is elevated to a height of 50 cm. Behavioural room was maintained at full light. Experimental sessions were videotaped by a camera fixed in front of the apparatus. Anxiety like behaviour was measured by placing the animals at the center of the plus-maze, facing the open arm. During a 10-min observation period, the following parameters were measured: number of open and closed arm entries, and percentage of time spent on open arms. Arm entry was counted when both rat forepaws were placed into the given arm. The % open arm time, an inverse measure of anxiety like behaviour, was calculated as (time in open arms/total time in arms) × 100.

### 4.6. Forced Swimming Test

The FST procedure was similar to that described by Porsolt et al. [30], except that the water was deeper [41]. The FST consisted of a two-day testing procedure. During the pretest session, rats were individually immersed for 15 min into a Plexiglass cylinder (70 cm high, 20 cm diameter) filled with 30 cm of water maintained at 25–27 °C. Twenty-four hours later, the same rats were exposed to a 5-min test session. At this depth of water in the cylinder, the animal was forced to either swim or float without its hind limbs or tail touching the bottom. Rats were moved and dried before returning them to home cages. Behavioural room was maintained at full light. Test session was video-recorded and the time that rats spent performing the following behaviours was measured: swimming (time spent making active swimming motion, e.g., moving around the cylinder), struggling (time spent in tentative of escaping making climbing or frenetic movements), drowning-recovering (time spent to remain under water, letting go, keeping the hind legs still the front upright followed by recovering), and immobility (time spent remaining afloat, performing the minimum amount of anterior extremity movement, simply to keep its head above the water). The duration was measured manually using a stopwatch. After pretest or test session the animal was dried with a paper towel and warmed under a heat lamp in home cage.

### 4.7. Statistics

All statistical analyses were performed using Graph Pad^®^ 6.0 for Windows. Data were tested for normality by the selection of parametric and non-parametric tests. Behavioural data were analysed by unpaired *t*-test, ordinary one-way or two-way analysis of variance (ANOVA), followed by Tukey Multiple Comparison’s test. Differences were considered significant only when *p*-value < 0.05.

## 5. Conclusions

The results obtained in this study indicate that BEO induces relaxant and anxiolytic effects with a different behavioural pattern compared to DZP and provide further insight to the neurobiological profile of the phytocomplex. However, further studies are needed to elucidate the correct mechanism of action of BEO to a rationale use in aromatherapy. Moreover, well designed clinical trials are needed to conclusively assess efficacy and tolerability of the phytocomplex for therapeutic use.
